# Capacity for brain amyloid PET in Germany: Results from the 1st survey on nuclear brain imaging in Germany

**DOI:** 10.1007/s00259-025-07237-8

**Published:** 2025-03-27

**Authors:** R. Buchert, A. Drzezga, M. Schreckenberger, P. T. Meyer, for the Working Group Nuclear Brain Imaging of the German Society of Nuclear Medicine (DGN e.V.)

**Affiliations:** 1 Department of Diagnostic and Interventional Radiology and Nuclear Medicine, University Medical Center Hamburg-Eppendorf, Martinistr. 52, 20246 Hamburg, Germany; 2 Institute of Neuroscience and Medicine (INM-2), Forschungszentrum Jülich, Jülich, Germany; 3 Department of Nuclear Medicine, Faculty of Medicine and University Hospital Cologne, University of Cologne, Köln, Germany; 4 German Center for Neurodegenerative Diseases (DZNE), Bonn-Cologne, Germany; 5 Department of Nuclear Medicine, Johannes Gutenberg University, Mainz, Germany; 6 Department of Nuclear Medicine, Medical Center - University of Freiburg, Freiburg, Germany

Dear Editor,

The decisive role of neuropathology biomarkers in the diagnosis of Alzheimer's disease (AD) [1–3] and the development of disease-modifying anti-amyloid AD therapies [4] present not only opportunities but also challenges for the nuclear medicine community. In Europe, there is a critical debate about whether there is sufficient availability, capacity and expertise for amyloid PET to be used in clinical practice to support patient selection for anti-amyloid therapies, treatment monitoring or the decision to discontinue treatment (when sufficient amyloid has been removed from the brain) [5, 6]. This is particularly true for countries like Germany, in which up to now there is no reimbursement by statutory health insurance and, consequently, no broadly established network of amyloid PET providers.

In a very recent editorial [7] presenting the EANM perspective on the expected approval of lecanemab (Leqembi™) in Europe [8], the authors recommended that “national societies for nuclear medicine … conduct a review of available and potential resources, in terms of scanning slots, personnel, and expertise” with regard to amyloid PET. Against this background, we report the results on the current status and the possibility of increasing the number of amyloid PET scans from the 1st survey on nuclear brain imaging in Germany, conducted from October 2023 to January 2024. A complete and detailed report of the entire survey will be published elsewhere.

For the survey, nuclear medicine physicians were contacted via email using the email lists of the German Society of Nuclear Medicine and the Professional Association of German Nuclear Medicine Physicians. A questionnaire was provided as a Microsoft Excel document attached to the email. It consisted of 33 questions divided into 3 parts: "Frequency & Indications": 20 questions, "Expertise & Acceptance": 7 questions, "Dopamine transporter SPECT methods": 6 questions. In order to limit the time to complete the questionnaire to 10 min, predefined response options were provided and participants were asked to respond by ticking one (or more) of the options. This also applied to questions about the frequency of certain imaging procedures, including (but not limited to) dopamine transporter (DAT) SPECT and amyloid PET, for which different ranges were provided as response options. Participants were asked to select the appropriate range "off the top of their head" (i.e., without searching databases). None of the questions required free-text responses. Participants were asked to return the completed questionnaire by e-mail.

A total of 85 completed questionnaires were received. Two questionnaires were excluded because they were copies from the same institution submitted by different persons. Another questionnaire was excluded because it was submitted by a non-German institution. The remaining 82 questionnaires were included: 33 (40%) from practices (PR), 15 (18%) from non-university community hospitals (CH) and 34 (42%) from university hospitals (UH) (Table 1). Thus, the vast majority of German UH participated in the survey. At the time of the survey, the Association of German University Hospitals represented 38 full members.

**Table 1:** Number of responding institutions across the German federal states both in absolute terms and per million inhabitants. (PR = practice or medical supply center, CH = non-university community hospital, UH = university hospital)

German federal state	PR (n)	CH (n)	UH (n)	Total (n)	Total per 1 million inhabitants
Baden-Württemberg	4	2	5	11	0.98
Bavaria	7	2	5	14	1.05
Berlin/Brandenburg	0	2	1	3	0.47
Bremen	1	0	0	1	1.46
Hamburg	2	0	1	3	1.59
Hesse	2	0	2	4	0.63
Mecklenburg-Vorpommern	0	0	2	2	1.23
Lower Saxony	2	1	2	5	0.61
North Rhine-Westphalia	10	5	9	24	1.32
Rhineland-Palatinate	2	1	1	4	0.96
Saarland	0	0	0	0	0.00
Saxony	3	2	2	7	1.71
Saxony-Anhalt	0	0	1	1	0.46
Schleswig–Holstein	0	0	2	2	0.68
Thuringia	0	0	1	1	0.47
Total	33	15	34	82	0.97

The reported frequency of DAT SPECT among the 82 responding institutions was used to assess the representativeness of the survey. DAT SPECT was performed in the vast majority (90%) of the responding institutions and with similar frequency in PR, CH and UH: on average about 100 scans per year. The total number of DAT SPECT scans reported by all responding institutions was about 7,800 per year. This represents 52% of all DAT SPECT examinations per year in Germany (n ≈ 14,890, based on data from the National Association of Statutory Health Insurance Physicians in Germany), suggesting that the survey is representative. In fact, the coverage of the current survey is well comparable to the survey on myocardial perfusion SPECT in Germany, which has been conducted since 2006 with very consistent results, covering 54% of all myocardial perfusion SPECT examinations in Germany in its latest run in 2022 (on 2021) [9].

Institutions were asked to indicate the number of clinical amyloid PET scans (independent of the tracer) annually performed at their institution by ticking one of the following categories: none, 1–5, 6–25, 26–50, 51–100 or > 100 scans. The results are shown in Fig. 1A. They suggest that the use of amyloid PET imaging is already relatively widespread, particularly at UH, with 76% of UH carrying out amyloid PET on a regular basis. In contrast, the majority of responding PR (82%) and CH (60%) reported that they do not perform amyloid PET. Overall, numbers of amyloid-PET examinations are currently still limited with only 12% of the responding institutions reporting to perform more than 50 amyloid PET scans per year. To estimate the total number of amyloid PET scans across all responding institutions, each of the predefined ranges was represented by its mean (e.g., 26–50 → (26 + 50)/2 = 38). The highest range was represented by 50% of the width of the second highest range above the cut-off (> 100 → 100 + (100–51)/2 = 125). In this way, the total number of clinical amyloid PET scans across all responding institutions was estimated to be approximately 800 per year. This does not include amyloid PET scans performed for research purposes.

**Fig. 1:**
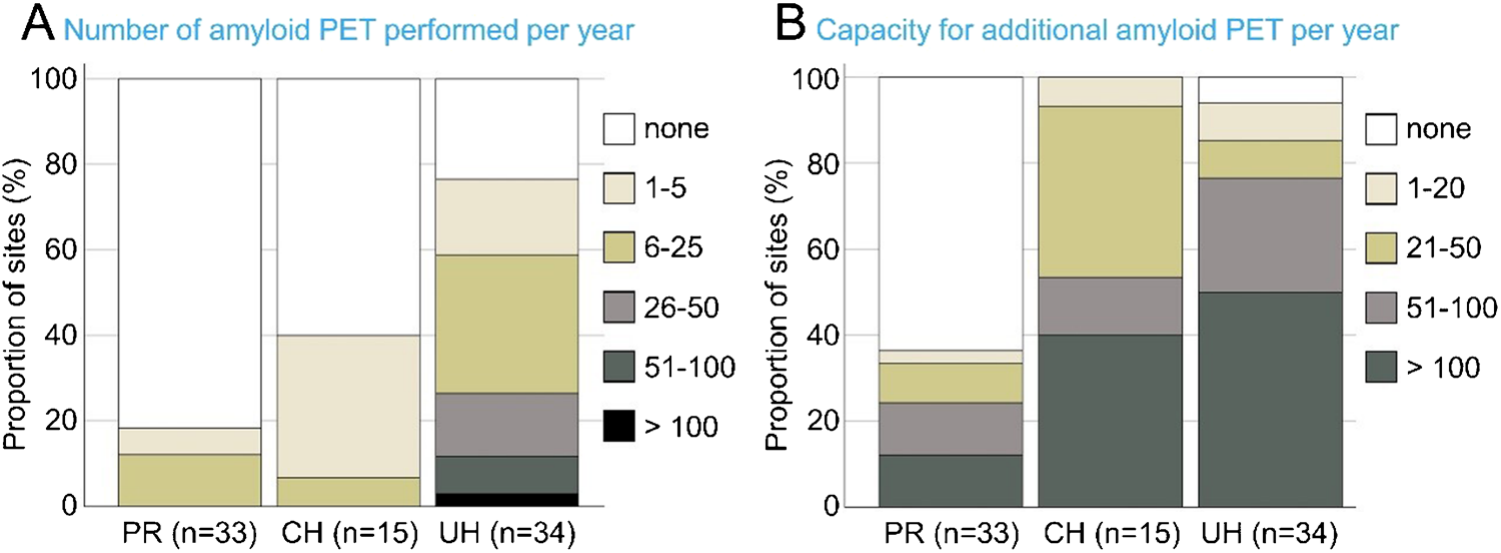
Number of amyloid PET scans performed per year at the 82 institutions that participated in the 1st survey of the status of nuclear brain imaging in Germany (**A**) and reported capacity per year for additional amyloid PET scans (**B**), assuming adequate reimbursement can be guaranteed. (PR = practice or medical supply center, CH = non-university community hospital, UH = University hospital)

Institutions were also asked to indicate their capacity to perform additional amyloid PET scans (assuming adequate reimbursement can be guaranteed) by ticking one of the following categories: none, 1–20, 21–50, 51–100, > 100 scans per year. The results are shown in Fig. 1B. The capacity for additional amyloid PET scans was more than 50 per year (1 per week) in 24%, 53% and 76% of the participating PR, CH and UH, respectively. The total additional capacity across all responding institutions (estimated analogous to the total number of amyloid PET scans currently performed) was almost 5,000 per year, indicating a capacity for an approximately sevenfold increase in the annual number of amyloid PET scans ((5,000 + 800)/800 ≈ 7).

In its recent report on the readiness of the Canadian health care system for the introduction of anti-amyloid AD therapies, Canada ‘s Drug Agency estimated that, in the first year after their approval, approximately 0.1% of the population aged 65 years and older may be referred to amyloid PET to assess eligibility for the novel treatment (the potential use of amyloid PET to monitor patients on anti-amyloid treatment was not taken into account) [10]. To obtain this estimate, Canada ‘s Drug Agency used a funnel approach with all persons aged 65 years or older as the starting population and made the following assumptions: (i) incident cases were used instead of prevalent cases to estimate the number of patients for capacity planning, assuming that prevalent AD cases would be too advanced to benefit from anti-amyloid therapies, (ii) per year, approximately 6% of the starting population will newly develop the underlying condition (MCI or dementia of any type), (iii) of these, approximately 20% will present to health services, (iv) of these, approximately 50% will be clinically diagnosed with early stage AD (MCI or mild dementia due to AD), (v) of these, approximately 60% will be assessed for eligibility for anti-amyloid therapy, and (vi) of these, approximately one third will be referred to amyloid PET, due to contraindication to lumbar puncture or borderline cerebrospinal fluid (CSF) results. Combining the assumed proportions results in the quoted 0.1% estimate (0.06*0.2*0.5*0.6*0.33 ≈ 0.001). In Germany, with about 18.7 million people aged 65 years and older (www.destatis.de, 2022), this would result in almost 19,000 amyloid PET examinations in the first year after approval of an anti-amyloid therapy. Canada’s drug agency projected a significant further increase in demand for amyloid PET over the following years [10].

Following the US Food and Drug Administration approval of aducanumab (Aduhelm™) in the summer of 2021, the EANM Neuroimaging Committee stated that “the nuclear medicine community, including tracer providers and imaging sites, projects an increase in the need of the amyloid PET as high as 20-fold and will have to guarantee a timely access to the technology and to ensure proper training of a large number of nuclear medicine physicians” [5, 6]. The prediction of a 20-fold increase in the demand for amyloid PET was based on the following assumptions: (i) proof of amyloid positivity will be required for a patient to be eligible for anti-amyloid treatment, (ii) after the introduction of blood amyloid markers into clinical practice, a two-step procedure with blood-based screening followed by confirmation with CSF testing or amyloid PET will be used, and (iii) approximately one third of patients requiring confirmation of amyloid positivity after blood-based screening will have contraindications to lumbar puncture, will refuse lumbar puncture or will have inconclusive CSF results, and will therefore be referred to amyloid PET [5]. In the current survey, the total number of clinical amyloid PET scans reported by all responding institutions was approximately 800 per year. An increase by a factor of 20 according to the EANM Neuroimaging Committee would result in approximately 16,000 amyloid PET scans, in reasonable agreement with the estimate of 19,000 amyloid PET scans based on the assumptions of Canada ‘s drug agency.

The capacity for a sevenfold increase of the number of amyloid PET examinations reported by the responding institutions of the German survey is less than the 20-fold increase in demand predicted by the EANM Neuroimaging Committee [5, 6]. However, the additional capacity reported by the participants of the survey refers to an immediate increase with existing PET/CT equipment. The survey does not take into account the potential for further capacity increases through the installation of additional scanners, e.g. dedicated brain PET scanners, or the replacement of existing scanners with new high-sensitivity scanners, which allow higher patient throughput by significantly reducing acquisition times. Furthermore, the number of patients referred to clinical anti-amyloid therapy in the US and other countries has not been excessive since the first compound was approved more than three years ago. For example, approximately 9,000 patients have been treated with lecanemab in the US since its accelerated approval in January 2023 [11]. In addition, the approval of currently available anti-amyloid therapies in Europe is likely to be limited to certain patient subgroups such as apolipoprotein E ε4 non-carriers or heterozygotes [8]. It is estimated that approximately 15% of all patients with AD are homozygous carriers of the E ε4-allele and therefore would not qualify for anti-amyloid therapy in Europe [12]. Finally, lumbar puncture and CSF testing are more established in Europe than in the US, and are often suggested as the first choice for amyloid status assessment, mainly because of lower costs [13]. Under these circumstances, the increase in demand for clinical amyloid PET scans may actually be smaller and slower than predicted.

Data obtained from the manufacturers of [^18^F]florbetaben (NeuraCeq™, Life Molecular Imaging) and [^18^F]flutemetamol (Vizamyl™, GE HealthCare) via the German Electrical and Electronic Manufacturers' Association (ZVEI) indicate that ≥ 400 physicians in Germany are trained to read amyloid PET with one or both of these tracers, with adequate coverage of all German federal states (Supplementary Table 1).

Sufficient coverage with commercially available amyloid radiotracers can also be assumed, as confirmed by the vendors and demonstrated by current multicenter-studies on amyloid-PET imaging such as the German coverage with evidence development study ENABLE [14].

Together these data suggest that the general conditions for increasing the number of amyloid PET scans are already in place in Germany: ubiquitous access to PET/CT scanners and commercial amyloid PET tracers, trained staff, and experience in performing and interpreting the test. The relatively low demand to date in part still affects the priority given to the commercial supply of amyloid radiotracers by providers. Some commercial providers offer radiotracers for amyloid imaging in certain regions only, on selected working days or at less favorable times (afternoon). To increase flexibility for the users, further improvement of the tracer availability is desirable and also to be expected due to the increasing demand in the future. The results of the survey also suggest that a large proportion of clinical amyloid PET scans are currently performed at UH. This must be interpreted with caution, as the survey included the vast majority of UH in Germany, but only a rather small proportion of PR and CH providing nuclear medicine services. However, it seems likely that the proportion of institutions performing amyloid PET is smaller among institutions that did not participate in the survey (e.g. because they do not perform nuclear brain imaging at all) than among those that did participate. It is therefore desirable that more PR and CH offer amyloid PET to broaden the base for clinical amyloid PET.

In conclusion, the cross-sectional self-reported data from the first survey on the status of nuclear brain imaging in Germany indicate that the availability, capacity and expertise for amyloid PET in Germany are generally sufficient to meet a higher demand for amyloid PET following approval of an anti-amyloid therapy. We believe that access to amyloid PET will therefore not be a major bottleneck for the introduction of new AD therapies into clinical practice. A further demand-driven expansion is expected (similar to what has been observed for PSMA-PET), provided that adequate reimbursement is ensured. Therefore, we believe that the discussion about appropriate diagnostic tools to assess eligibility for novel anti-amyloid therapies should not be driven by the argument of perceived lack of access to amyloid PET. More generally, the results of the survey may be helpful in the expected discussion between healthcare providers/physicians, the pharmaceutical industry, health insurance providers, government authorities and patients/-organizations, and may facilitate forward-looking planning of upcoming therapies. The German survey may motivate other European countries to carry out similar surveys in the near future.

## Supplementary Information

Below is the link to the electronic supplementary material.

(DOCX 48.2 kb)

## Data Availability

Not applicable.
