# Supplementary material for: Capacity for brain amyloid PET in Germany: Results from the 1st survey on nuclear brain imaging in Germany
Source: Eur J Nucl Med Mol Imaging. 2025 Mar 27;52(10):3613–7. doi: 10.1007/s00259-025-07237-8 (PMC12316748; doi:10.1007/s00259-025-07237-8)
Supplement: Supplementary file 1 — (DOCX 48.2 kb) [file 259_2025_7237_MOESM1_ESM.docx]

**Supplementary Material**

**Capacity for brain amyloid PET in Germany: Results from the 1^st^ Survey on Nuclear Brain Imaging in Germany**

R. Buchert^1,*^, A. Drzezga^2,3,4,*^, M. Schreckenberger^5^ and P.T. Meyer^6^ , for the Working Group Nuclear Brain Imaging of the German Society of Nuclear Medicine (DGN e.V.)

^1^Department of Diagnostic and Interventional Radiology and Nuclear Medicine, University Medical Center Hamburg-Eppendorf, Hamburg, Germany

^2^Institute of Neuroscience and Medicine (INM-2), Forschungszentrum Jülich, Germany

^3^Department of Nuclear Medicine, Faculty of Medicine and University Hospital Cologne, University of Cologne, Germany

^4^German Center for Neurodegenerative Diseases (DZNE), Bonn-Cologne, Germany

^5^Department of Nuclear Medicine, Johannes Gutenberg University, 55101 Mainz, Germany.

^6^Department of Nuclear Medicine, Medical Center - University of Freiburg, Freiburg, Germany

^*^These authors contributed equally as first authors

**Corresponding author:** Ralph Buchert, Department of Diagnostic and Interventional Radiology and Nuclear Medicine, University Medical Center Hamburg-Eppendorf, Martinistr. 52, 20246 Hamburg, Germany, Email: r.buchert@uke.de, Phone: +49 (0)40 7410-54347, Fax: +49 (0)40 7410-40265, ORCID ID 0000-0002-0945-0724

**Supplementary Table 1** Number of physicians trained for the reading of amyloid-PET scans with [^18^F]florbetaben or [^18^F]flutemetamol per 1 million inhabitants. Physicians trained for both tracers were counted twice. (Source: German Electrical and Electronic Manufacturers' Association ZVEI, 03/2024).

| German federal state | Number of trained physicians  per 1 million inhabitants |
| --- | --- |
| Baden-Württemberg | 3.9 |
| Bavaria | 7.0 |
| Berlin/Brandenburg | 10.1 |
| Bremen | 4.4 |
| Hamburg | 5.3 |
| Hesse | 3.6 |
| Mecklenburg-Vorpommern | 6.8 |
| Lower Saxony | 4.4 |
| North Rhine-Westphalia | 6.3 |
| Rhineland-Palatinate | 3.8 |
| Saarland | 22.2 |
| Saxony | 2.4 |
| Saxony-Anhalt | 7.3 |
| Schleswig-Holstein | 4.1 |
| Thuringia | 3.3 |
| Total | 5.7 |
